# Early warning system using primary health care data in the post-COVID-19 pandemic era: Brazil nationwide case-study

**DOI:** 10.1590/0102-311XEN010024

**Published:** 2024-12-20

**Authors:** Thiago Cerqueira-Silva, Juliane F. Oliveira, Vinicius de Araújo Oliveira, Pilar Tavares Veras Florentino, Alberto Sironi, Gerson O. Penna, Pablo Ivan Pereira Ramos, Viviane S. Boaventura, Manoel Barral-Netto, Izabel Marcilio

**Affiliations:** 1 Instituto Gonçalo Moniz, Fundação Oswaldo Cruz, Salvador, Brasil.; 2 Centro de Medicina Tropical, Universidade de Brasília, Brasília, Brasil.

**Keywords:** COVID-19, Syndromic Surveillance, Respiratory Diseases, COVID-19, Vigilância Sindrômica, Doenças Respiratórias, COVID-19, Vigilancia Sindrómica, Enfermedades Respiratorias

## Abstract

Syndromic surveillance using primary health care (PHC) data is a valuable tool for early outbreak detection, as demonstrated by the potential to identify COVID-19 outbreaks. However, the potential of such an early warning system in the post-COVID-19 era remains largely unexplored. We analyzed PHC encounter counter of respiratory complaints registered in the database of the Brazilian Unified National Health System from October 2022 to July 2023. We applied EARS (variations C1/C2/C3) and EVI to estimate the weekly thresholds. An alarm was determined when the number of encounters exceeded the week-specific threshold. We used data on hospitalization due to respiratory disease to classify as anomalies the weeks in which the number of cases surpassed predetermined thresholds. We compared EARS and EVI efficacy in anticipating anomalies. A total of 119 anomalies were identified across 116 immediate regions during the study period. The EARS-C2 presented the highest early alarm rate, with 81/119 (68%) early alarms, and C1 the lowest, with 71 (60%) early alarms. The lowest true positivity was the EARS-C1 118/1,354 (8.7%) and the highest was EARS-C3 99/856 (11.6%). Routinely collected PHC data can be successfully used to detect respiratory disease outbreaks in Brazil. Syndromic surveillance enhances timeliness in surveillance strategies, albeit with lower specificity. A combined approach with other strategies is essential to strengthen accuracy, offering a proactive and effective public health response against future outbreaks.

## Resumo

A vigilância sindrômica utilizando dados de atenção primária à saúde (APS) é uma ferramenta valiosa para a detecção precoce de surtos, conforme demonstrado no potencial de identificar surtos de COVID-19. No entanto, o potencial desse sistema de alerta antecipado na era pós-COVID-19 continua amplamente inexplorado. Foram analisadas as contagens de atendimentos na APS por queixas respiratórias registradas na base de dados do Sistema Único de Saúde de outubro de 2022 a julho de 2023. O EARS (variações C1/C2/C3) e o EVI foram aplicados para estimar os limiares semanais. Um alarme foi criado para quando o número de atendimentos excedesse o limite específico da semana. Dados de hospitalização por doença respiratória foram utilizados para classificar semanas em que o número de casos ultrapassou os limites predeterminados como anomalias. Comparamos a eficácia do EARS e do EVI na antecipação de anomalias. Um total de 119 anomalias foram identificadas em 116 regiões imediatas durante o período do estudo. O EARS-C2 apresentou a maior taxa de alarmes precoces, com 81 de 119 (68%) alarmes precoces, enquanto o C1 apresentou a menor, com 71 (60%) alarmes precoces. A menor taxa de verdadeiros positivos foi a EARS-C1 118/1.354 (8,7%) e a maior EARS-C3 99/856 (11,6%). Os dados de APS coletados rotineiramente podem ser usados com sucesso para detectar surtos de doenças respiratórias no Brasil. A vigilância sindrômica melhora a prontidão das estratégias de vigilância, embora com menor especificidade. Uma abordagem combinada com outras estratégias é essencial para fortalecer a precisão, oferecendo uma resposta proativa e eficaz de saúde pública contra futuros surtos.

## Resumen

La vigilancia sindrómica, que utiliza datos de la atención primaria de salud (APS), es una herramienta valiosa para la detección temprana de brotes, como lo demuestra el capacidad para identificar brotes de COVID-19. Sin embargo, el uso de este sistema de alerta temprana en la era posterior a la COVID-19 sigue en gran medida inexplorado. Se analizaron los conteos de atenciones en la APS por afecciones respiratorias registradas en la base de datos del Sistema Único de Salud desde octubre del 2022 hasta julio del 2023. Se aplicaron el EARS (rangos C1/C2/C3) y el EVI para estimar los umbrales semanales. Se emitió una alarma en caso de que el número de atenciones excediera el límite específico para la semana. Los datos de hospitalización por enfermedad respiratoria se utilizaron para clasificar como anómalas las semanas en las que el número de casos superó los umbrales predeterminados. Comparamos la eficacia del EARS y del EVI para anticipar anomalías. Durante el período del estudio se identificó un total de 119 anomalías en 116 regiones. El EARS-C2 presentó la tasa más alta de alarmas tempranas, con 81 de 119 (68%) alarmas tempranas, mientras que el C1 tuvo la más baja, con 71 (60%) alarmas tempranas. La tasa de positividad más baja fue la EARS-C1 118/1.354 (8,7%) y la más alta EARS-C3 99/856 (11,6%). Los datos de APS recopilados de forma rutinaria pueden utilizarse con éxito para detectar brotes de enfermedades respiratorias en Brasil. La vigilancia sindrómica aumenta la prontitud de las estrategias de vigilancia, aunque con menor especificidad. Un enfoque combinado con otras estrategias es esencial para fortalecer la precisión y proporcionar una respuesta de salud pública proactiva y eficaz contra futuros brotes.

## Introduction

The COVID-19 pandemic has underscored the importance of timely and accurate surveillance systems in detecting and responding to emerging infectious diseases ^1^ ^,^ ^2^. Traditional surveillance methods, such as relying on laboratory-confirmed cases and hospital data, have timeliness, representativeness, and coverage limitations ^3^ ^,^ ^4^. Moreover, traditional surveillance systems do not prioritize early warning, and their usefulness for early detection of outbreaks has not been established ^5^.

Syndromic surveillance systems were implemented to help provide situational awareness and inform patterns of illness distribution ^6^ ^,^ ^7^. Syndromic surveillance relies on a set of pre-defined diagnostic symptoms, which are available before laboratory pathogen identification, therefore adding timeliness and sensitivity to the surveillance system. In this sense, integrating primary health care (PHC) data into syndromic surveillance systems is regarded as a valuable source of information for early outbreak detection ^8^ ^,^ ^9^.

The advancements in technology and the increasing availability of routinely collected health data highlight the importance of integrating digital health approaches to establish early warning systems for pandemic readiness and response ^1^ ^,^ ^2^. The use of digital health in early warning enables the collection and analysis of diverse data streams. It represents a cost-effective solution by using data routinely gathered for healthcare and administrative purposes.

In the context of influenza-like illness (ILI), the importance of syndromic surveillance at the PHC level is particularly pronounced as the number of individuals with severe respiratory disease seeking emergency rooms is expected to rise a few weeks after a marked increase in mild cases seeking PHC assistance. Brazil, with its vast population and comprehensive publicly funded healthcare system ^10^, provides an ideal setting to evaluate the potential of digital syndromic surveillance for anticipating ILI outbreaks. A previous study demonstrated the capabilities of using PHC data for the early detection of the COVID-19 first wave ^11^.

This study aims to evaluate the potential of digital syndromic surveillance using PHC data for respiratory diseases to establish an early warning system in the post-COVID-19 pandemic era.

## Methods

### Study design

We evaluated the capabilities of an early warning system based on the weekly updated national PHC database using data on hospitalizations due to respiratory diseases as a gold standard. The study period ranged from October 15, 2022 to July 29, 2023. All analyses were aggregated by the geographic immediate region according to the Brazilian Institute of Geography and Statistics (IBGE, acronym in Portuguese) ^11^.

### Data source

PHC data: the Brazilian Health Information System for Primary Care (SISAB, acronym in Portuguese) contains data on all publicly funded PHC encounters in the country, coded by either the International Classification of Diseases - 10th revision (ICD-10) or the International Classification of Primary Care - 2nd edition (ICPC-2). The PHC system covers at least 75% of the population in Brazil ^12^. Data for PHC encounters were extracted from the SISAB database, obtained under the permission of the Brazilian Ministry of Health. We used weekly counts of every PHC encounter due to ILI from October 2022 to July 2023. We included 50 ICD-10 and ICPC-2 codes corresponding to conditions possibly related to ILI (Supplementary Material - Table S1; https://cadernos.ensp.fiocruz.br/static//arquivo/suppl-e00010024_6750.pdf).

Hospital information system: the Brazilian Hospital Information System (SIH, acronym in Portuguese) comprises information on all publicly funded hospitalizations in Brazil, coded by the ICD-10. Data were extracted from October 15, 2022 to July 29, 2023. We included 24 ICD-10 codes corresponding to respiratory conditions (Supplementary Material - Table S2; https://cadernos.ensp.fiocruz.br/static//arquivo/suppl-e00010024_6750.pdf).

### Statistical methods

We compared two methods to determine the threshold for an early warning in the PHC time series: the Early Aberration Reporting System (EARS, variations C1/C2/C3) ^13^ and the Epidemic Volatility Index (EVI) ^14^. The stark differences observed in the PHC time series between the years pre- and post-COVID-19 resulted in a nonstable baseline; thus an early warning system in the post-COVID-19 era required anomaly detection methods suitable for working with very short time series. The EARS method was developed by the U. S. Centers for Disease Control and Prevention (CDC) to operate with short time series, requiring as little as three time points ^13^. The EVI was developed by Kostoulas et al. ^14^ based on calculating the rolling standard deviation for a time series of confirmed COVID-19 cases and can also be employed with a short time series.

We used an 8-week baseline for EARS and EVI, with a 0.05 threshold alpha for the EARS and a 0.1 threshold c for the EVI. The baseline of an 8-week period is calculated differently for the C1 and C2/C3 variations. In the case of C1, the baseline uses data from the weeks -1 to -8, while C2/C3 uses data from weeks -3 to -10 ^15^. In this scenario, we only start to determine thresholds for all methods in January 2023 (the 11th week after October 15, 2022).

Additionally, we conducted two sensitivity analyses: (1) changing the threshold alpha of 0.01 for EARS and c 0.2 for EVI to test the effect in specificity; and (2) using a 4-week baseline to test the effect in timeliness. An alarm in the PHC-based early warning system is established when the current number of encounters surpasses the week-specific threshold.

We defined an anomaly in the SIH time series by comparing the number of hospitalizations in the current week with the median number of hospitalizations per week in the study period. The thresholds for each immediate region were defined considering the median number of hospitalizations (Supplementary Material - Box S1; https://cadernos.ensp.fiocruz.br/static//arquivo/suppl-e00010024_6750.pdf). Anomalies separated by one week alone were merged into one single event. Anomalies lasting for only one week were discarded, this criterion was applied to distinguish genuine events from random variation.

We evaluated the PHC-based early warning performance using three metrics derived from Nekorchuk et al. ^16^: (1) percent of events recorded, defined as the percent of anomalies in the SIH time series recorded by the PHC-based early warning; (2) percent of alarms associated with an anomaly (true positives): an alarm and anomaly were considered associated if the alarm was triggered any week during or up to three weeks prior to the anomaly; (3) percent of timely alarms: defined as an alarm up to three weeks prior to the first week of an anomaly. We conducted the analysis stratified by population size of the immediate region, categorized as small, medium, or large, as defined by the first and third quartiles. The weighted PHC coverage of each immediate region was calculated as the mean coverage of the municipalities comprising that region divided by the municipality population.

The statistical analysis was conducted using R software, version 4.3.1 (http://www.r-project.org), and the packages *surveillance* and *EVI*.

### Ethics aspects

The study is based on secondary, aggregated, non-identified data, and was approved by the Research Ethics Committee of Oswaldo Cruz Foundation - Brasília Regional Office (CAAE: 61444122.0.0000.0040).

## Results

**Figure 1:**
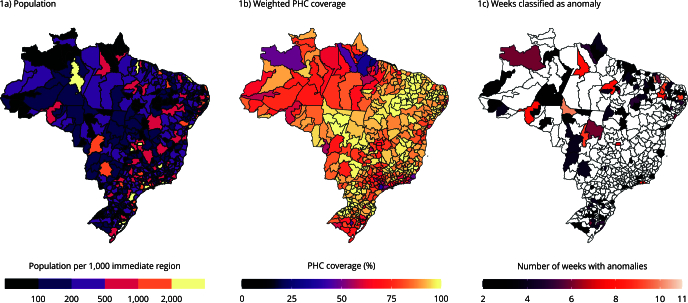
Brazil’s immediate regions. From January to July, 2023.

Brazil has 510 immediate regions with populations ranging from 30,000 to 20 million inhabitants. The median population per region is 185,349 (interquartile range - IQR: 117,752; 324,635) (Figure 1). The weighted PHC coverage per immediate region ranged from 27% to 100%, with a median of 91% (IQR: 80; 97) (Figure 1).

PHC: primary health care.

**Figure 2:**
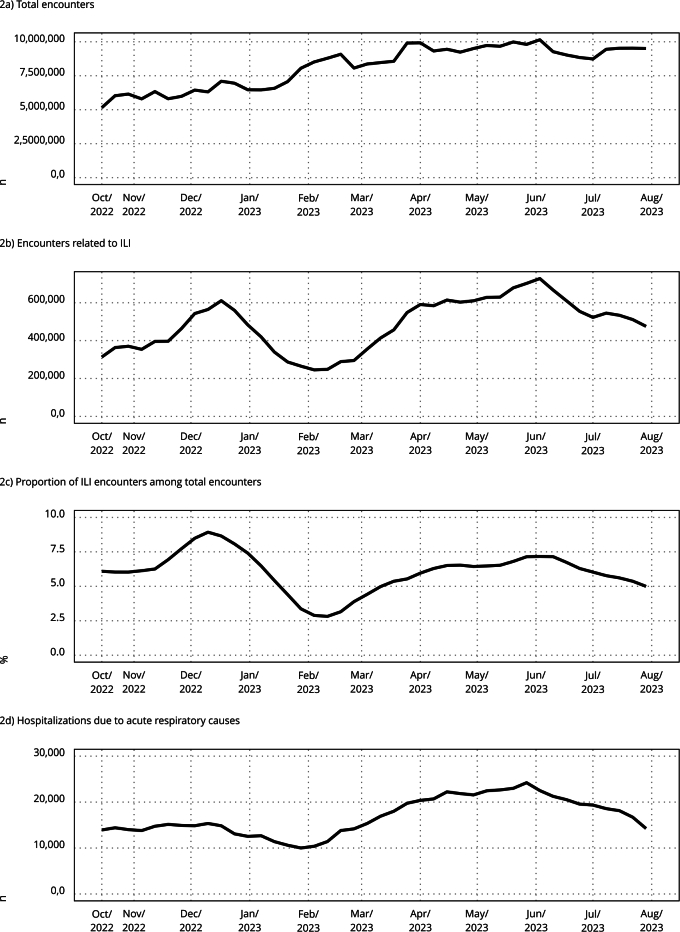
Encounters in the primary health care (PHC) and hospitalizations due to acute respiratory causes (4-week moving average). Brazil, October 2022 to July 2023.

The total number of PHC encounters steadily increased during the study period, peaking close to 10,000,000 encounters per week in June of 2023. The number of ILI-related encounters and hospitalizations due to respiratory causes also peaked around June 2023 (Figure 2).

ILI: influenza-like illness.

**Figure 3:**
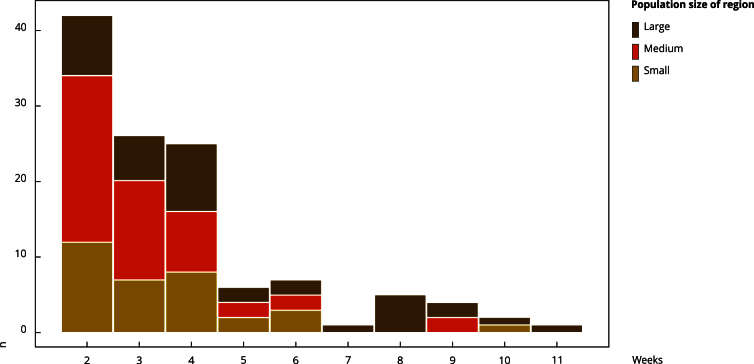
Extent of anomalies stratified by population size.

**Figure 4:**
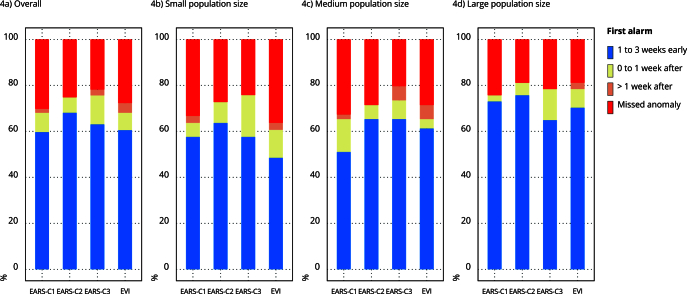
Performance of the methods, considering the time (in weeks) of the first alarm in the primary health care (PHC) series in relation to the first week with an anomaly in the Brazilian Hospital Information System (SIH) series, overall and stratified by population size.

EARS: Early Aberration Reporting System; EVI: Epidemic Volatility Index.

We identified 119 anomalies across 116 immediate regions in the SIH time series from January to July 2023, lasting from 2 to 11 weeks (Figures 1 and 3). The EARS-C2 presented the highest early alarm rate in the PHC time series, with 81/119 (68%) early alarms, and C1 presented the lowest, with 71 (60%) early alarms (Figure 4; Table 1); 52 (44%) anomalies were early detected across all three variations of EARS and EVI, and 15 anomalies (13%) were not detected in any method (Table 2). Most missed anomalies in all methods lasted only two weeks (Table 3). The true positivity was similar for all methods, ranging from 9% (EARS-C1) to 12% (EARS-C3) (Table 4).

**Table 1:** Performance of the methods, considering the time (in weeks) of the first alarm in the primary health care (PHC) series in relation to the first week with an anomaly in the Brazilian Hospital Information System (SIH) series, overall and stratified by population size.

First Alarm	Overall (N = 119) [n (%)]				Small (N = 33) [n (%)]				Medium (N = 49) [n (%)]				Large (N = 37) [n (%)]			
	EARS-C1	EARS-C2	EARS-C3	EVI	EARS-C1	EARS-C2	EARS-C3	EVI	EARS-C1	EARS-C2	EARS-C3	EVI	EARS-C1	EARS-C2	EARS-C3	EVI
1 to 3 weeks early	71 (59.7)	81 (68.1)	75 (63.0)	72 (60.5)	19 (57.6)	21 (63.6)	19 (57.6)	16 (48.5)	25 (51.0)	32 (65.3)	32 (65.3)	30 (61.2)	27 (73.0)	28 (75.7)	24 (64.9)	26 (70.3)
0 to 1 week after	10 (8.4)	8 (6.7)	15 (12.6)	9 (7.6)	2 (6.1)	3 (9.1)	6 (18.2)	4 (12.1)	7 (14.3)	3 (6.1)	4 (8.2)	2 (4.1)	1 (2.7)	2 (5.4)	5 (13.5)	3 (8.1)
> 1 week after	2 (1.7)	0 (0.0)	3 (2.5)	5 (4.2)	1 (3.0)	0 (0.0)	0 (0.0)	1 (3.0)	1 (2.0)	0 (0.0)	3 (6.1)	3 (6.1)	0 (0.0)	0 (0.0)	0 (0.0)	1 (2.7)
Missed anomaly	36 (30.3)	30 (25.2)	26 (21.8)	33 (27.7)	11 (33.3)	9 (27.3)	8 (24.2)	12 (36.4)	16 (32.7)	14 (28.6)	10 (20.4)	14 (28.6)	9 (24.3)	7 (18.9)	8 (21.6)	7 (18.9)

EARS: Early Aberration Reporting System; EVI: Epidemic Volatility Index.

**Table 2:** Number of anomalies that multiple methods detected early or missed.

Number of methods	Anomalies detected early [n (%)]	Anomalies missed [n (%)]
0	22 (18.5)	76 (63.9)
1	14 (11.8)	6 (5.0)
2	16 (13.4)	7 (5.9)
3	15 (12.6)	15 (12.6)
4	52 (43.7)	15 (12.6)

**Table 3:** Extent of missed anomalies by method.

Weeks	EARS-C1 (N = 36) [n (%)]	EARS-C2 (N = 30) [n (%)]	EARS-C3 (N = 26) [n (%)]	EVI (N = 33) [n (%)]
2	15 (42.0)	14 (47.0)	10 (38.0)	15 (45.0)
3	8 (22.0)	5 (17.0)	7 (27.0)	9 (27.0)
4	8 (22.0)	7 (23.0)	5 (19.0)	6 (18)
5	2 (5.6)	2 (6.7)	1 (3.8)	2 (6.1)
6	3 (8.3)	2 (6.7)	2 (7.7)	1 (3.0)
8	0 (0.0)	0 (0.0)	1 (3.8)	0 (0.0)

EARS: Early Aberration Reporting System; EVI: Epidemic Volatility Index.

**Table 4:** True positive rates per method.

Population size	EARS-C1 [n/N (%)]	EARS-C2 [n/N (%)]	EARS-C3 [n/N (%)]	EVI [n/N (%)]
Overall	118/1,354 (8.7)	135/1,185 (11.4)	99/856 (11.6)	115/1,051 (10.9)
Small	28/345 (8.1)	32/305 (10.5)	27/232 (11.6)	25/270 (9.3)
Medium	43/679 (6.3)	55/581 (9.5)	40/425 (9.4)	44/531 (8.3)
Large	47/330 (14.2)	48/299 (16.1)	32/199 (16.1)	46/250 (18.4)

EARS: Early Aberration Reporting System; EVI: Epidemic Volatility Index.

In the stratified analysis by population size, immediate regions with small populations had the lowest early alarms rate, ranging from 48% to 64%, while regions with large populations had the highest rate, with values ranging 65%-76% (Figure 4). The true positivity rates were low across all strata, slightly better in large population regions, with values from 14 to 18% (Table 4).

The sensitivity analysis using the 0.01 threshold alpha for the EARS method and *c* 0.2 for EVI did not increase the true positive rate. However, the early detection rate decreased for all methods, ranging from 36% (C1) to 56% (C2) (Tables 5 and 6). The analysis using 4-week baseline improved the early detection rate for EARS (C2 and C3) and EVI, increasing from 68 to 72% (C2), 63 to 72% (C3), and 61 to 71% (EVI), and improved caught rate in the same methods. The true positive rate decreased by 1 or 2% in all methods (Tables 5 and 6).

**Table 5:** Performance of the methods in the two sensitivity analyses.

First alarm	Sensitivity - change threshold values (N = 119) [n (%)]				Sensitivity - change baseline value (N = 119) [n (%)]			
	EARS-C1	EARS-C2	EARS-C3	EVI	EARS-C1	EARS-C2	EARS-C3	EVI
1 to 3 weeks early	43 (36.1)	67 (56.3)	63 (52.9)	56 (47.1)	58 (48.7)	86 (72.3)	86 (72.3)	85 (71.4)
0 to 1 week after	14 (11.8)	10 (8.4)	15 (12.6)	11 (9.2)	18 (15.1)	6 (5.0)	10 (8.4)	8 (6.7)
> 1 week after	2 (1.7)	2 (1.7)	4 (3.4)	4 (3.4)	2 (1.7)	2 (1.7)	3 (2.5)	8 (6.7)
Missed anomaly	60 (50.4)	40 (33.6)	37 (31.1)	48 (40.3)	41 (34.5)	25 (21.0)	20 (16.8)	18 (15.1)

EARS: Early Aberration Reporting System; EVI: Epidemic Volatility Index.

Note: N represents the total of anomalies.

**Table 6:** True positive rates per method of the two sensitivity analyses.

Characteristic	Sensitivity - change threshold values [n (%)]				Sensitivity - change baseline value [n (%)]			
	EARS-C1 (N = 882)	EARS-C2 (N = 955)	EARS-C3 (N = 792)	EVI (N = 808)	EARS-C1 (N = 1,806)	EARS-C2 (N = 1,500)	EARS-C3 (N = 1,064)	EVI (N = 1,467)
True positive	73 (8.3)	105 (11.0)	87 (11.0)	82 (10.1)	112 (6.2)	136 (9.1)	117 (11.0)	129 (8.8)

EARS: Early Aberration Reporting System; EVI: Epidemic Volatility Index.

Note: N represents the total of anomalies.

## Discussion

This study outcomes provide valuable insights into the longitudinal patterns of encounters due to respiratory causes in the PHC and the use of PHC data to develop an early warning system for respiratory disease outbreaks. We employed two methods for the early warning: EARS and EVI. Both methods showed capacity for early detection of respiratory disease outbreaks, with overall detection rate ranging 60%-68%. However, population size impacted the true positivity and early detection rate, with small population regions presenting the lowest number of true positives and early alarms.

The EARS and EVI methods offer flexibility for use in situations with limited historical data by relying on recent information for threshold setting. However, they exhibit a notable drawback: a decreased ability to accommodate seasonality, resulting in alarms often triggered during seasonal peaks. Methods able to adjust for seasonality, such as the improved Farrington method, tend to perform better in the true positive metric ^17^. The sustained high fluctuations of PHC encounters due to COVID-19 cases from 2020 to 2022, with misleading low numbers during lockdown periods, hinder the use of methods that require longer historical data as a baseline ^18^ ^,^ ^19^. Bédubourg & Le Strat ^17^ compared 21 early warning methods using simulated datasets and found that the probability of detection of an outbreak ranged from 43.3% to 84.4%, and the false positive rate ranged from 0.7% to 59.9%. The EARS variations showed a probability of detection ranging from 54.2% to 68% and a false positive rate of 6.9% to 8.5%. Similar to our findings, the C2 EARS variation presented the best performance metrics. The authors concluded that no single method presented outbreak detection performances sufficient enough to provide reliable monitoring for a large surveillance system ^17^. Using real surveillance data, Nekorchuk et al. ^16^ compared three early warning methods for detecting malaria outbreaks. They found that the improved Farrington method showed the most effective results, as it could achieve the best trade-off on maximizing both sensitivity (> 70%) and specificity (> 70%). Similar to our study, when analyzing the three EARS variations, they found a high percentage of events caught (80% to 100%), with a moderate early detection rate (43% to 87%) and a low true positive rate (25% to 40%) ^16^.

In the context of an article on early warning systems, it is important to highlight the distinct advantages of integrating PHC data with conventional surveillance systems ^8^ ^,^ ^9^. This is particularly relevant in Brazil, where the granularity of PHC is exceptionally valuable. PHC extends its reach even to regions that lack more advanced healthcare facilities, reaching underserved rural and remote regions ^10^. The granularity of PHC plays a pivotal role in offering a timely window for detecting alarms by syndromic surveillance. It enables the early recognition of emerging health threats, even in areas with limited access to higher complexity healthcare infrastructure. This, in turn, allows for more rapid responses and proactive public health measures, ultimately enhancing the resilience of the healthcare system and safeguarding the well-being of vulnerable populations ^20^.

The performance metrics shown here should be interpreted within the context of syndromic surveillance early alarms. It is generally accepted that syndromic surveillance provides high sensitivity but low specificity ^6^, and that the usefulness of early warning systems resides in indicating that an aberrant situation is occurring and should be investigated by health authorities ^21^. Different parameterizations of detection methods will yield different performances, and, in general, a trade-off between the power of detection, false positive rates, and early detection should be considered when choosing a particular method ^16^ ^,^ ^22^ ^,^ ^23^. The choice of which method to use depends on the data availability and which detection characteristics are most important in a given situation ^24^.

This study is part of the Alert-Early System for Outbreaks with Pandemic Potential (ÆSOP) ^25^. This system is intended to supplement traditional surveillance methods used by the Brazilian Ministry of Health, which are based on mandatory notification of suspected cases. Furthermore, our system relies on the use of existing information systems in order to achieve cost-effectiveness and avoid adding to the already overburdened health care system ^25^. In Brazil (2023), 89% of public healthcare institutions use electronic health record systems, with 61% using the Brazilian Ministry of Health system (*Prontuário Eletrônico do Cidadão*) ^26^. This scenario enables the quick transfer of data over a unified system. Given that the infrastructure for data collection and storage is already in place as part of the health-care routine, implementing the PHC-based warning system would be substantially less expensive than building new data-capture infrastructure. On the other hand, we anticipate that investing in training health surveillance personnel is critical for increasing capacity. Continuous education is required to maintain a qualified team capable of interpreting information from various sources and planning activities to validate warning signals at different administrative levels. A recent systematic review on the effectiveness of early warning systems indicated that syndromic surveillance is more proactive to detect outbreaks. However, it presented mixed results in terms of the accuracy of the outbreak detection ^27^.

Our study presents potential limitations. First, we relied on an algorithmic approach using reported hospitalizations due to acute respiratory causes to as a gold standard for defining outbreaks. Thus, respiratory diseases that do not progress to severe disease would not be considered an outbreak in this study, and the signal detected in the PHC data would be considered a false positive. Second, we could not link individual PHC encounters and SIH data, preventing us from estimating the percentage of individuals who first sought PHC before developing severe symptoms. Third, the PHC data encompass only encounters in the public health sector. This is a potential bias in settings with a higher percentage of usage of the private health sector. For instance, outbreak detection could be less timely if an outbreak started among the wealthier stratum of the population. Furthermore, we did not have access to laboratory data, which would have provided specificity to the early warning, favoring reaction and mitigation actions.

In conclusion, this study highlights the value of leveraging digital syndromic surveillance for the early detection of outbreaks. It offers valuable insights into using routinely collected PHC data for respiratory disease outbreak detection in Brazil. Working in this endeavor is crucial for enhancing surveillance accuracy and mitigating future outbreaks. This study contributes to the growing body of knowledge essential for addressing the complex challenges posed by infectious diseases, thus promoting a more proactive and effective public health response.
