# Supplementary material for: Early warning system using primary health care data in the post-COVID-19 pandemic era: Brazil nationwide case-study
Source: Cad Saude Publica. 2024 Dec 20;40(11):e00010024. doi: 10.1590/0102-311XEN010024 (PMC11654108; doi:10.1590/0102-311XEN010024)
Supplement: Supplementary file 1 [file 1678-4464-csp-40-11-EN010024-s.pdf]

SUPPLEMENTARY MATERIAL

Table S1 ICD-10 and ICPC-2 codes used to define conditions likely related to influenza-like illness.

Type	Code	Description
ICPC-2	A03	Fever
ICPC-2	R01	Pain respiratory system
ICPC-2	R02	Shortness of breath/dyspnoea
ICPC-2	R03	Wheezing
ICPC-2	R04	Breathing problem, other
ICPC-2	R05	Cough
ICPC-2	R07	Sneezing / nasal congestion
ICPC-2	R08	Nose symptom / complaint other
ICPC-2	R21	Sinus symptom / complaint
ICPC-2	R23	Voice symptom / complaint
ICPC-2	R25	Sputum / phlegm abnormal
ICPC-2	R29	Respiratory symptom/complaint other
ICPC-2	R71	Whooping cough
ICPC-2	R74	Upper respiratory infection acute
ICPC-2	R75	Sinusitis acute/chronic
ICPC-2	R76	Tonsillitis acute
ICPC-2	R77	Laryngitis/tracheitis acute
ICPC-2	R78	Acute bronchitis/bronchiolitis
ICPC-2	R80	Influenza
ICPC-2	R81	Pneumonia
ICPC-2	R83	Respiratory infection other
ICPC-2	R99	Respiratory disease other
ICD-10	J00	Acute nasopharyngitis
ICD-10	J01	Acute sinusitis
ICD-10	J02	Acute pharyngitis
ICD-10	J03	Acute tonsillitis
ICD-10	J04	Acute laryngitis and tracheitis
ICD-10	J06	Acute upper respiratory infections of multiple and unspecified sites
ICD-10	J09	Influenza due to identified zoonotic or pandemic influenza virus
ICD-10	J10	influenza due to identified seasonal influenza virus
ICD-10	J11	Influenza, virus not identified
ICD-10	J12	Viral pneumonia, not elsewhere classified
ICD-10	J13	Pneumonia due to Streptococcus pneumoniae
ICD-10	J14	Pneumonia due to Haemophilus influenzae
ICD-10	J15	Bacterial pneumonia, not elsewhere classified
ICD-10	J16	Pneumonia due to other infectious organisms, not elsewhere classified
ICD-10	J17	Pneumonia in diseases classified elsewhere
ICD-10	J18	Pneumonia, organism unspecified
ICD-10	J20	Acute bronchitis
ICD-10	J21	Acute bronchiolitis
ICD-10	J22	Unspecified acute lower respiratory infection
ICD-10	J80	Adult respiratory distress syndrome
ICD-10	R05	Cough
ICD-10	R06	Abnormalities of breathing
ICD-10	R07	Pain in throat and chest
ICD-10	R43	Disturbances of smell and taste
ICD-10	R50	Fever of other and unknown origin
ICD-10	U07	Emergency use of U07
ICD-10	B34	Viral infection of unspecified site
ICD-10	B97	Viral agents as the cause of diseases classified to other chapters

Table S2 ICD-10 codes used to define hospitalisations due to respiratory causes.

Type	Code	Description
ICD-10	J00	Acute nasopharyngitis
ICD-10	J01	Acute sinusitis
ICD-10	J02	Acute pharyngitis
ICD-10	J03	Acute tonsillitis
ICD-10	J04	Acute laryngitis and tracheitis
ICD-10	J06	Acute upper respiratory infections of multiple and unspecified sites
ICD-10	J09	Influenza due to identified zoonotic or pandemic influenza virus
ICD-10	J10	influenza due to identified seasonal influenza virus
ICD-10	J11	Influenza, virus not identified
ICD-10	J12	Viral pneumonia, not elsewhere classified
ICD-10	J13	Pneumonia due to Streptococcus pneumoniae
ICD-10	J14	Pneumonia due to Haemophilus influenzae
ICD-10	J15	Bacterial pneumonia, not elsewhere classified
ICD-10	J16	Pneumonia due to other infectious organisms, not elsewhere classified
ICD-10	J17	Pneumonia in diseases classified elsewhere
ICD-10	J18	Pneumonia, organism unspecified
ICD-10	J20	Acute bronchitis
ICD-10	J21	Acute bronchiolitis
ICD-10	J22	Unspecified acute lower respiratory infection
ICD-10	J80	Adult respiratory distress syndrome
ICD-10	U07	Emergency use of U07
ICD-10	B34	Viral infection of unspecified site
ICD-10	B97	Viral agents as the cause of diseases classified to other chapters
ICD-10	U04	Severe acute respiratory syndrome

Box S1 Additional methods.

Definition of an anomaly occurrence was defined as a function of the median (p50) of hospitalisations due to acute respiratory causes from January to July 2023. The rules for anomaly threshold are summarised in the table below. For handling random fluctuations in regions with a p50 less than 50 hospitalisations, the definition of an anomaly occurrence in a given week required a minimum of 10 hospitalisations in that week.	
**Median (p50) of hospitalisations due to respiratory causes**	**Threshold**
p50<50	N > (2 * p50) & N>10
50 ≤ p50 <100	N > (p50 + 0.5*p50)
100 ≤ p50 <250	N > (p50 + 0.4*p50)
250 ≤ p50 <500	N > (p50 + 0.3*p50)
500 ≤ p50 <1000	N > (p50 + 0.2*p50)
p50≥1000	N > (p50 + 0.1*p50)
